# Novel and effective synthesis protocol of AgNPs functionalized using L-cysteine as a potential drug carrier

**DOI:** 10.1007/s00210-017-1440-x

**Published:** 2017-11-16

**Authors:** Marek Wojnicki, Magdalena Luty-Błocho, Magdalena Kotańska, Magdalena Wytrwal, Tomasz Tokarski, Anna Krupa, Marcin Kołaczkowski, Adam Bucki, Marcin Kobielusz

**Affiliations:** 1 Faculty of Non-Ferrous Metals, AGH University of Science and Technology, Al. A. Mickiewicza 30, 30-059 Krakow, Poland; 2 Department of Pharmacological Screening, Chair of Pharmacy, Jagiellonian University, Medical College, 9 Medyczna Street, 30-688 Krakow, Poland; 3 Academic Centre for Materials and Nanotechnology, AGH University of Science and Technology, al. A. Mickiewicza 30, 30-059 Krakow, Poland; 4 Department of Pharmaceutical Technology and Biopharmaceutics, Jagiellonian University Medical College, 9 Medyczna Street, 30-688 Krakow, Poland; 5 Department of Medicinal Chemistry, Jagiellonian University, Medical College, 9 Medyczna Street, 30-688 Krakow, Poland; 6 Faculty of Chemistry, Jagiellonian University in Kraków, ul. Ingardena 3, 30-060 Krakow, Poland

**Keywords:** Silver nanoparticles, Functionalization, l-cysteine, Toxicity, Drug carrier, Synthesis

## Abstract

In this study, the protocol of a single-step l-cysteine functionalized silver nanoparticle synthesis was described. Particle size distribution was determined. The crystallinity and chemical properties were investigated using XRD, HR-TEM, and XPS methods. Acute toxicity and irritant properties of obtained nanoparticles were studied using mice and rats as an animal model. The results showed that thanks to the applied protocol, it was possible to synthesize silver nanoparticles with narrow particle size distribution. Moreover, the concentration of final product was extremely high in comparison to other known methods. These nanoparticles showed neither irritant properties nor acute toxicity.

## Introduction

Throughout the last decades, the nanotechnology has been considered as a powerful field, which finds out the application in many areas of science. The progress of nanotechnology is especially important in clinical medicine, where development of new drugs, devices, and diagnostic tools improves our life. In medical application, particular noble metals, such as gold, platinum, palladium, and silver nanoparticles are considered, due to their unique chemical and physical properties. These particles have low toxicity, high compatibility to biological systems, and can be easily functionalized, which increase the range of their potential application. For these reasons, gold nanoparticles are used, e.g., in cancer therapy mainly as drug carriers (Cobley et al. 2011), delivery vehicles (Galdiero et al. 2011), or markers for imaging (Huang et al. 2006) etc. (Guo and Sadler 1999) Due to their antibacterial properties, silver nanoparticles have been used mostly in healthcare industry, food storage, textile manufacturing, cosmetics (Kokura et al. 2010), and environmental remediation. It is worth noting, however, that the application of nanoparticles is strictly dependent on their morphology (size, shape, and their size distribution), which can be easily manipulated by the use of a suitable manufacturing technique. For example, chemical methods allow to synthetize nanoparticles of different morphology (Luty-Błocho et al. 2014; Luty-Błocho and Wojnicki 2015). Among them, only a few synthesis routes are recommended for medical application as there are problems with the biocompatibility of reagents, or media, etc. Thus, in the present study, chemical reduction of metal precursor with reductant was chosen as a method for the synthesis of silver nanoparticles. The main advantages of this technique are low cost, simplicity, and the opportunity to synthetize the nanoparticles which size around 9 nm. Dimethylamine borane was used as an effective reducing agent of silver ions (Wojnicki et al. 2015). In order to exclude a negative effect coming from products of nanoparticle synthesis, the solution after redox reaction was dialyzed (see the “Silver nanoparticle synthesis and characterization” section for more details). This method allows to remove all dangerous agents. In this paper, the special attention was focused on the functionalization of silver nanoparticles for cosmetic application.

## Materials and methods

### Silver nanoparticle synthesis and characterization

Silver nanoparticle synthesis was performed using silver(I) nitrate(V) (AgNO_3_, Avantor Materials) as precursor. Borane dimethylamine complex of 97% (DMAB, Fluka) was used as the reducing agent. l-cysteine was used as stabilizer as well as functionalizing compound. AgNPs were synthesized as follows: 0.2 M aqueous solution of AgNO_3_ was mixed in a 1:1 ratio with the aqueous solution containing 0.4 M of l-cysteine and 1 M of sodium hydroxide (Avantor Materials). Such a solution has lightly yellow color due to the formation of l-cysteine-Ag^+^ complex. Then, freshly prepared aqueous solution containing 0.3 M DMAB in 0.1 M NaOH was mixed in a 1:1 volumetric ratio with l-cysteine-Ag^+^ containing solution. After mixing, the color of the solution was gradually changing from light yellow by red to dark brown.

It is worth to mention here that increase of DMAB initial concentration affects the decrease of particle size. However, there is no linear correlation between reductant concentration and particle size.

The dialysis method was applied to remove the residues after redox reaction. For this purpose, the Medical International Size 9 Int. Dia. 36/32″ dialysis tubing was applied. The dialysis was performed for ca. 5 days. The continuous water exchange was set at 20 mL/min. Thanks to that, the pH level decreased to ca. 7. Also, the specific odor of dimethylamine was removed. Then, the solution was filtered using a Whatman PC MB membrane of 47 mm in diameter and the pore size of 50 nm. This solution was used for further analysis and experiments.

Zeta potential as well as particle size distribution of the obtained composite material was determined using a Zetasizer Nano ZS (Malvern). A standard clear polycarbonate cell with gold electrodes was applied.

UV-Vis spectra were registered using a Shimadzu model U-2501 PC and quartz cuvette of 10 mm path length.

Fluorescence spectra were measured using a PerkinElmer LS55 spectrofluorometer in a 1 cm quartz cuvette.

HR-TEM analysis was performed using HR-TEM—FEI TECNAI TF 20 X-TWIN. One drop of freshly prepared colloidal suspension was placed on a copper grid covered with a 20–30-nm amorphous carbon film. Then, the sample was left to dry at room temperature (ca. 21 °C).

Particle size and size distribution were determined after analyzing of HR-TEM images using ImageJ application (ver. 1.48b).

X-ray photoelectron spectroscopy measurements were performed using a PHI 5000 Versa Probe II (ULVAC-PHI, Chigasaki, Japan) system using a microfocused (100 μm, 25 W) Al Kα X-ray beam with a photoelectron take-off angle of 45°. To compensate the charging effect, a dual-beam charge neutralizer was used. The operating pressure in the analytical chamber was less than 5 × 10^−7^ Pa. High-resolution spectra were collected with analyzer pass energy of 23.50 eV. XPS spectra were acquired from 400 × 300 μm (Galdiero et al. 2011) areas. All XPS peaks were referenced to the neutral (C-C) carbon C 1s peak at 284.8 eV. Spectrum background was subtracted using the Shirley method. Data analysis software from PHI MultiPak was used to calculate elemental compositions from the peak areas.

### Animal studies

Adult female Albino Swiss mice weighing 28–33 g were used in the study. They were kept in environmentally controlled rooms, in standard cages lit by an artificial light for 12 h. Animals had free access to food and water. The randomly established experimental groups consisted of six mice.

Male Wistar rats of initial body weight from 200 to 230 g were also used. The animals were housed in plastic cages in constant temperature facilities exposed to 12–12 light-dark cycle. Water and food were available ad libitum. The solution of nanoparticles was administered on the shaved skin of the abdomen.

The solution of nanoparticles was administered intraperitoneally (i.p.)—group no. 1 or intragastrically by means of an oral gavage (p.o.)—group no. 2. The volume of the test solution was 10 mL/kg. Nothing was given to the control group—group no. 3.

All animal care and experimental procedures were carried out in accordance with European Union and Polish legislation acts concerning animal experimentation and approved by the Local Ethics Committee at the Jagiellonian University in Krakow.

### Acute toxicity, plasma, and tissue collection

After administration of tested solution, mice were placed into the home cages and observed. The percent of death and body weight changes were determined during 24 h. Subsequently, heparin (500 j/mice) and thiopental (70 mg/kg i.p.) for each animal was administrated. After 20 min, blood was collected from left common carotid artery. The animal spinal cord was interrupted, cut, and organs, namely the liver, kidneys, heart, brain, and lungs were weighted to determine the amount of nanosilver.

### Topical toxicity and skin collection

Thiopental (70 mg/kg i.p.) was administrated to each animal. After 20 min, the abdominal skin was smeared with depilatory cream (Joanna Sensitive, Poland), and 10 min later, the skin was wiped using a cotton swab moistened with 0.9% saline. The solution of nanoparticles (0.3 ml/animals) was administered on the skin. The percent of death and body weight changes were determined during 24 h. Subsequently, thiopental (70 mg/kg i.p.) was administrated to each animal. After 20 min, the skin was collected.

### Statistical analysis

The results obtained were analyzed using a two-way analysis of variance (ANOVA), followed by a Bonferroni post-hoc test with the significance level set at 0.05 (body weight changes). The outcomes were expressed as the means ± standard error of the mean (SEM). GraphPad Prism 6.0 was used for data analysis.

### Metal concentration determination

The biodistribution of AgNPs was investigated in selected organs. For this purpose, after 24 h, the liver, kidneys, heart, brain, and lungs were collected to determine the amount of nanosilver. Then, these organs were weighted and mineralized to extract silver. The extraction was performed according to protocol described in our previous papers (Wojnicki et al. 2013; Bednarski et al. 2015). The metal concentration was performed using spectrometer a 4200MP-AES Agilent.

## Results

### AgNP characterization

**Fig. 1:**
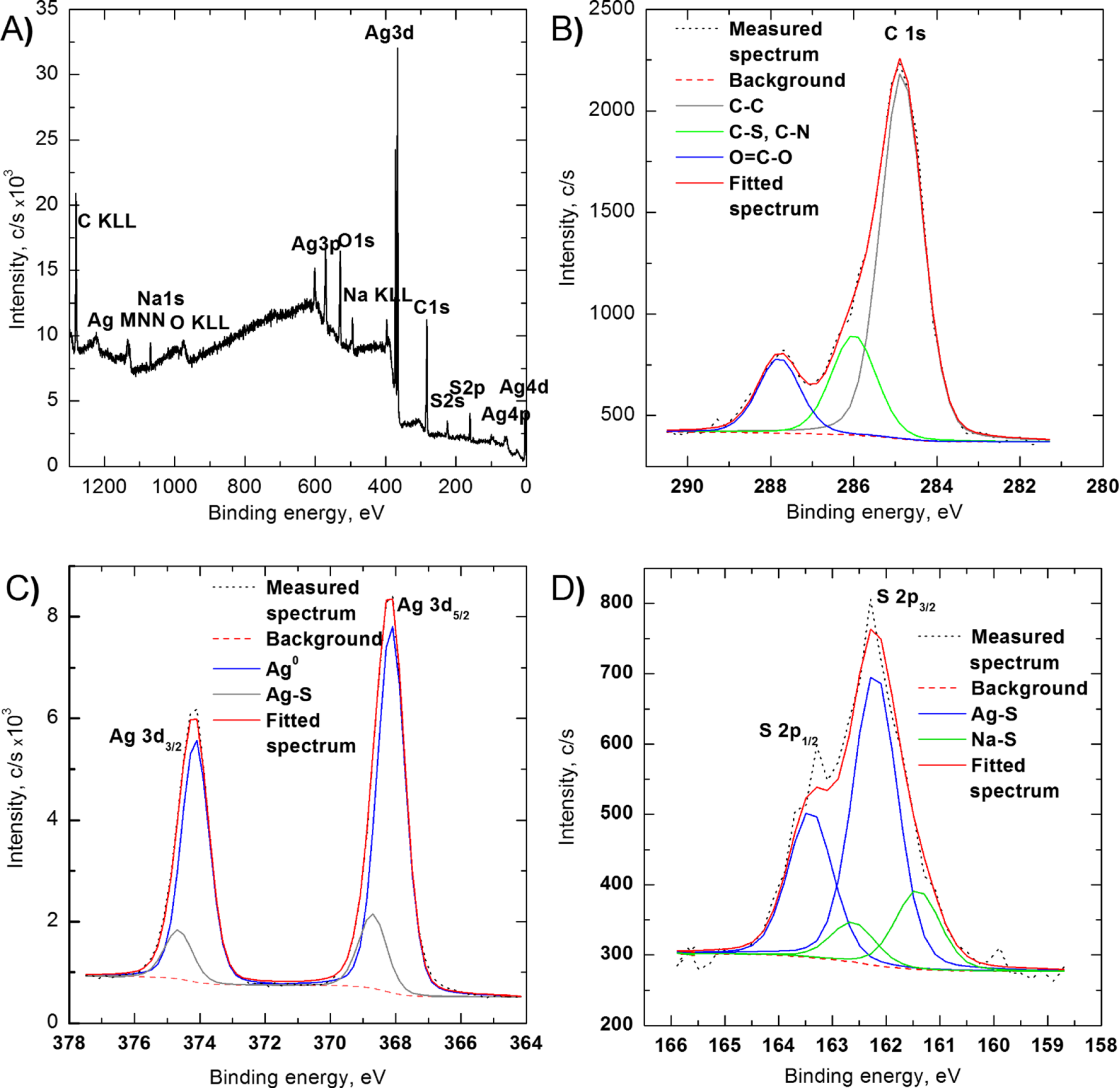
Survey scan performed with X-ray photoelectron spectroscopy of AgNPs (**a**). XPS spectra with fitted lines C 1s (**b**), Ag 3d (**c**), and S 2p (**d**)

X-ray photoelectron spectroscopy (XPS) of AgNPs confirmed the presence of silver nanoparticles with cysteine conjugated to the surfaces of the nanoparticles. The survey scan also indicated the presence of Ag, O, C, S, N, and Na elements in the sample (Fig. 1a). The atomic concentrations of each element were determined to be as the following: C 1s—54.56%; N 1s—6.05%; O 1s—21.29%; S 2p—5.24%; Ag 3d—10.71%; and Na 1s—2.15%. Detailed analysis of selected elements and bound type in AgNPs is shown in Fig. 1b–d. Figure 1b shows spectrum of C 1s with fitted peaks that come from cysteine bounded to nanoparticle surfaces: peak at 286.0 eV corresponds to C-N and C-S bonds while at 287.8 eV comes from O-C=O group in cysteine structure (Cavalleri et al. 2001). Ag 3d spectrum (Fig. 1c) can be fitted by two doublets with binding energies of the Ag 3d5/2 component at 368.2 and 368.7 eV (DS = 6.0 eV), respectively. First peak is attributed to metallic Ag, while the second is attributed to Ag-S from nanoparticle-cysteine bonds (Tong et al. 2015; Ferraria et al. 2012). These results confirmed that cysteine was chemically bonded to AgNP surfaces. Figure 1d shows S 2p spectrum. Peak deconvolution revealed presence of two chemical states of sulfur at 161.4 and 162.6 eV for S 2p3/2. The first corresponds to Na-S bond formed by cysteine in alkaline solution. This suggests that cysteine could be also physically adsorbed on the particle surfaces. The second peak comes from Ag-S bonds between particle and cysteine (Battocchio et al. 2014).

**Fig. 2:**
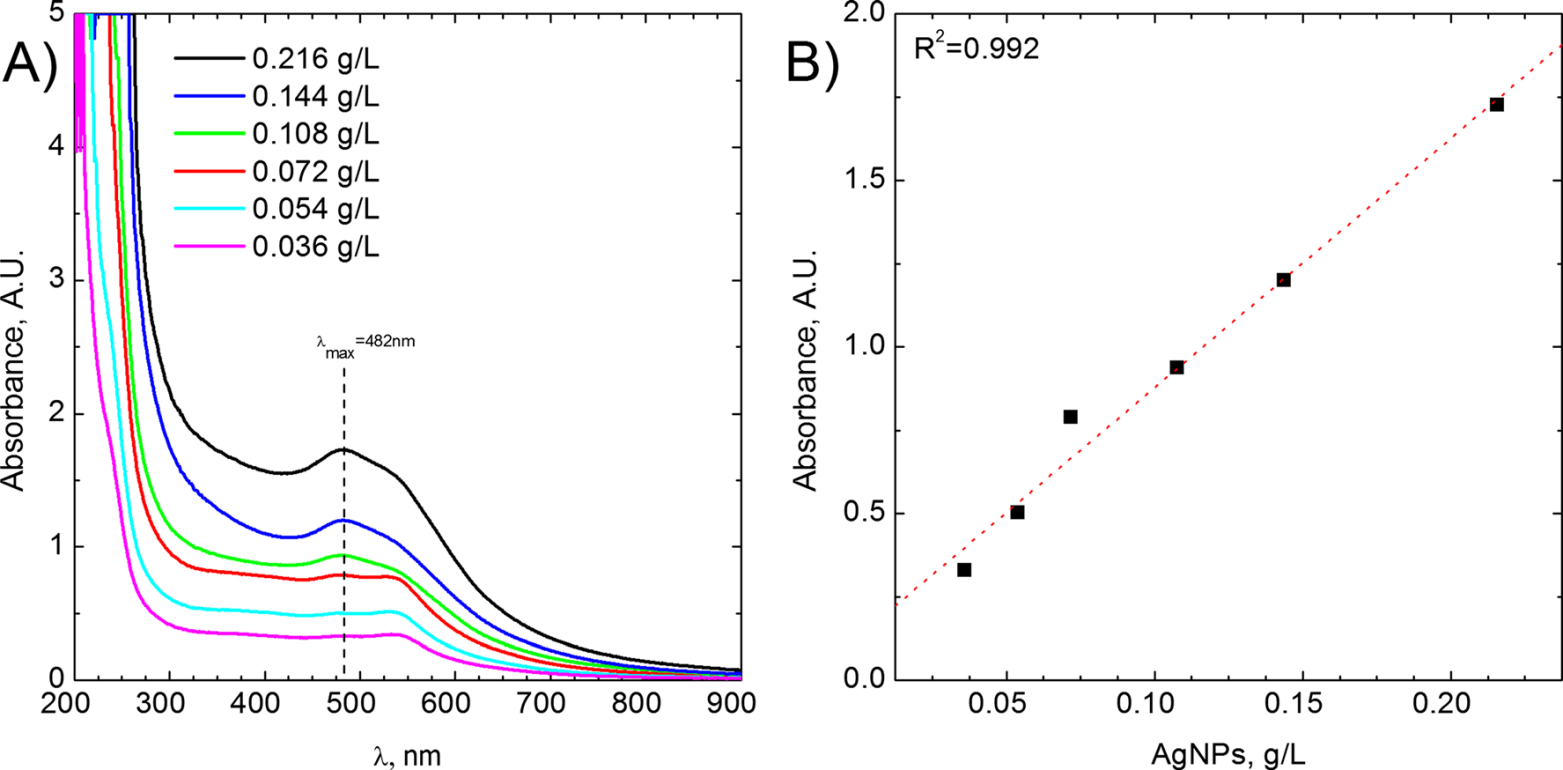
**a** UV-Vis spectrum of obtained AgNPs. **b** Determination of absorption coefficient

It is well known that the silver sulfide is a semiconductor, and its presence in the system should be observable in the UV-Vis spectra. In the case of AgNPs, their presence can also be confirmed thanks to plasmon resonance peak. Therefore, UV-Vis analyses were performed, and results obtained are shown in Fig. 2a.

In Fig. 2a, UV-Vis spectra of the solutions are shown. It has to be underlined that these spectra were recorded for diluted samples. The samples were diluted from 50 to 300 times, respectively. As it can be seen, one asymmetrical peak at the wavelength of 482 nm can be observed. This peak can be attributed to surface plasmon resonance (Darroudi et al. 2010). The red shift of plasmon resonance might be related to l-cysteine molecule on the surface of AgNPs (Knauer et al. 2013). It should be noted that UV-Vis analysis did not confirm the presence of Ag_2_S.

The absorption coefficient of synthetized colloid can be calculated taking into account the assumption that the absorbance is a linear function of the colloid concentration, according to Lambert-Beer’s law. From the slope of colloid concentration vs. absorbance, the absorption coefficient can be determined (see Fig. 2b). The slope is equal to 7.49 ± 0.48 $\frac{L}{\mathrm{g}\times \mathrm{cm}}$, and the intercept is 0.13 ± 0.06.

**Fig. 3:**
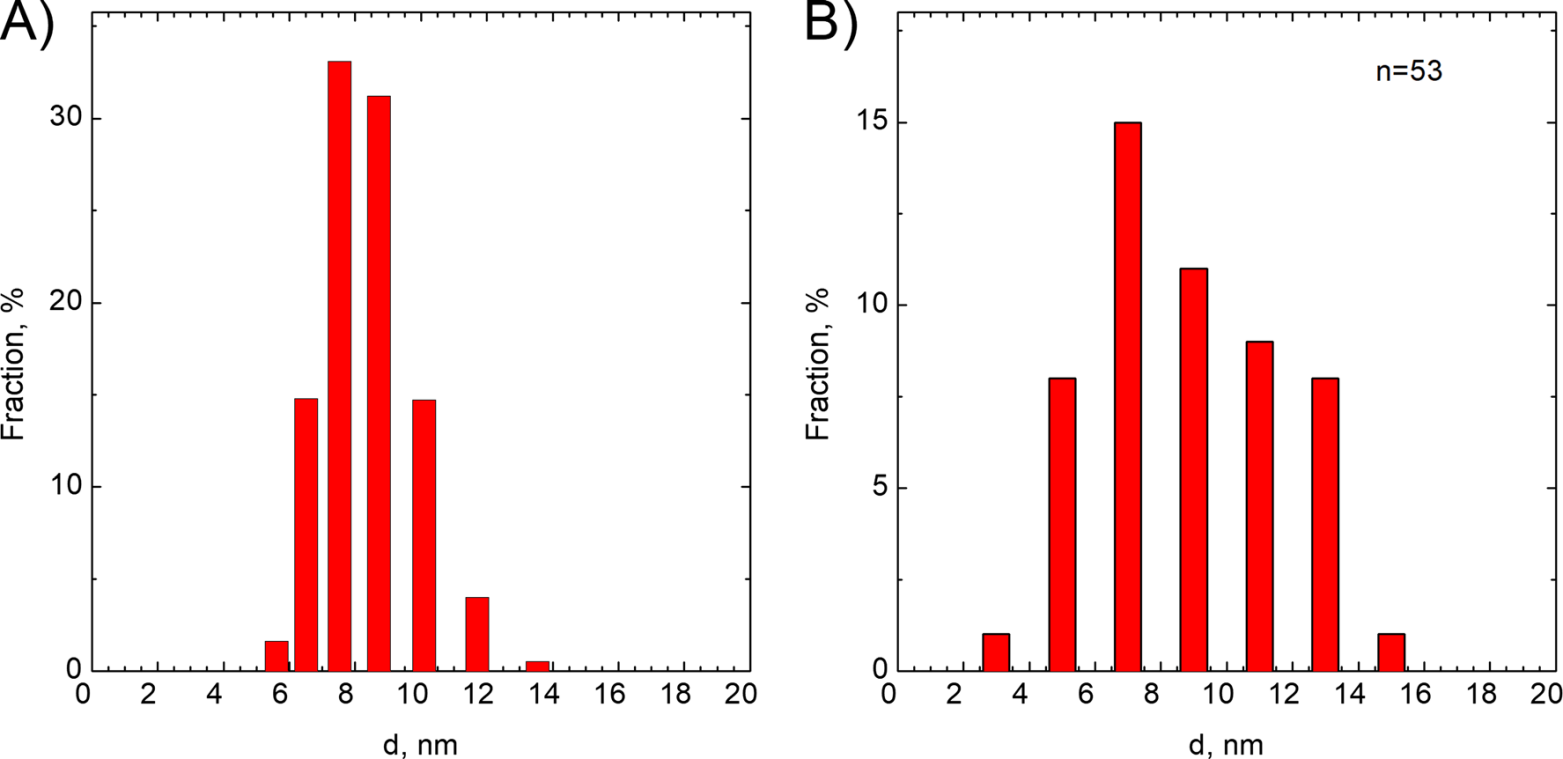
Particle size distribution determined using **a** DLS method and **b** HR-TEM image analysis method

Particle size and size distribution as well as zeta potential were determined using a Malvern instrument. The zeta potential determined using this equipment is equal to − 50.6 ± 4.47 mV. Strong negative zeta potential is probably related to the zwitterionic properties of l-Cysteine (Quesada-Moreno et al. 2014). Deprotonation of l-cysteine depends on pH. Two forms are present in the system at a pH of 7. The first one is the zwitterion and anionic form. Moreover, using DLS method, particle size and size distribution were determined. The results are shown in Fig. 3a. To make it clear, in Fig. 3b, particle size distribution determined using HR-TEM images is also shown.

As it can be seen, the main fraction is of about 8 nm in diameter using DLS as well as HR-TEM image analysis method. It should also be noted that the distribution of the particle size is symmetrical, whereas typical methods of nanoparticle synthesis result in log-normal particle size distribution. In case of HR-TEM image analysis method, particle size distribution seems to be wider and is equal to ca. 3.2 nm. During HR-TEM image analysis, 53 particles were analyzed (*n* = 53).

**Fig. 4:**
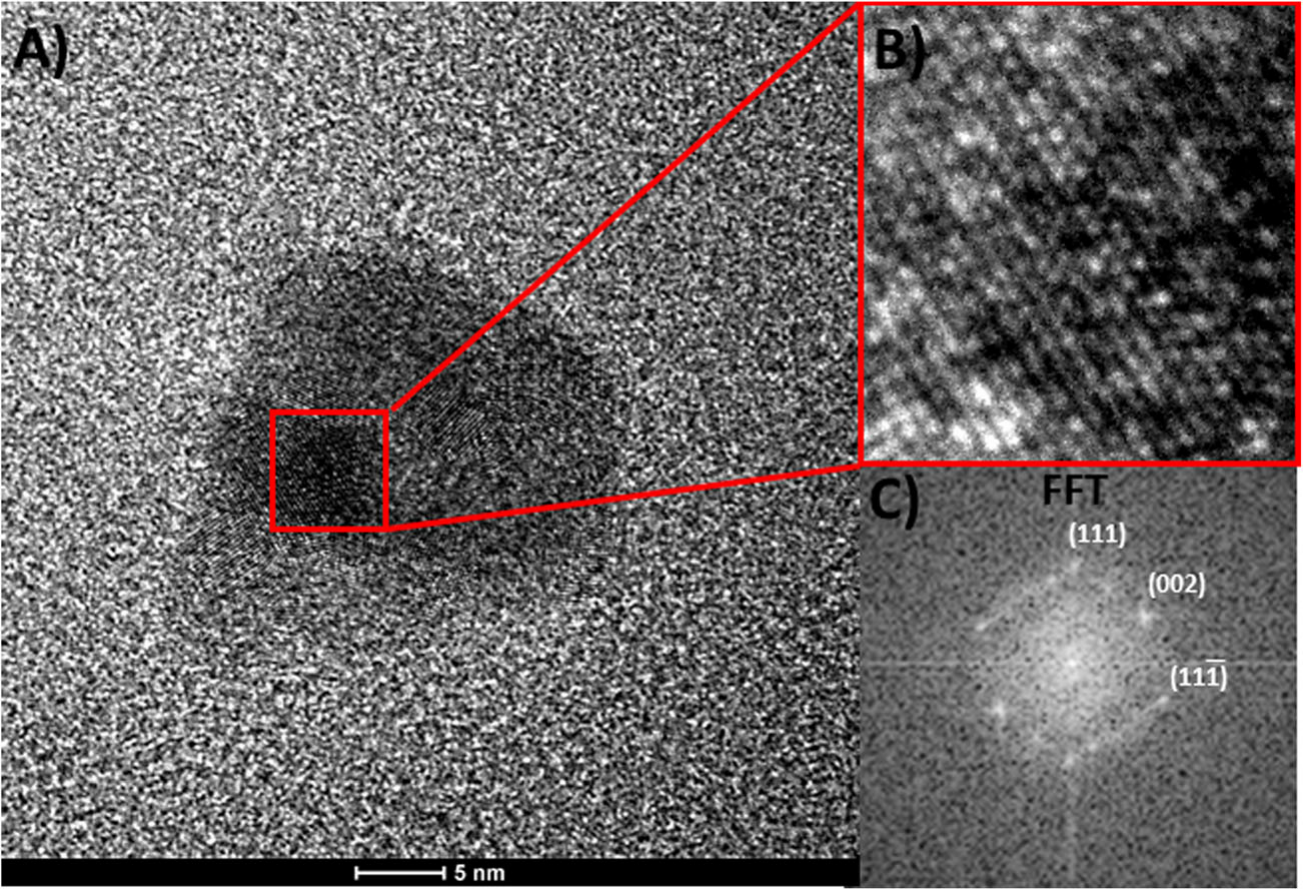
HR-TEM analysis (**a**) and selection of polycrystal structure (**b**) FFT analysis of single crystal (**c**)

The HR-TEM analysis was performed (Fig. 4a); the particle size was comparable to that determined using DLS method.

Moreover, FFT analysis of a selected picture (red square in Fig. 4a) was performed to obtain the information about crystallography of synthetized material (Fig. 4c). Selected crystal plane are marked on the diffractogram. The angle between planes (111) and (002) is equal to 54°, which is in agreement to the literature. However, it should be underlined that obtained nanoparticles have polycrystalline structure.

**Fig. 5:**
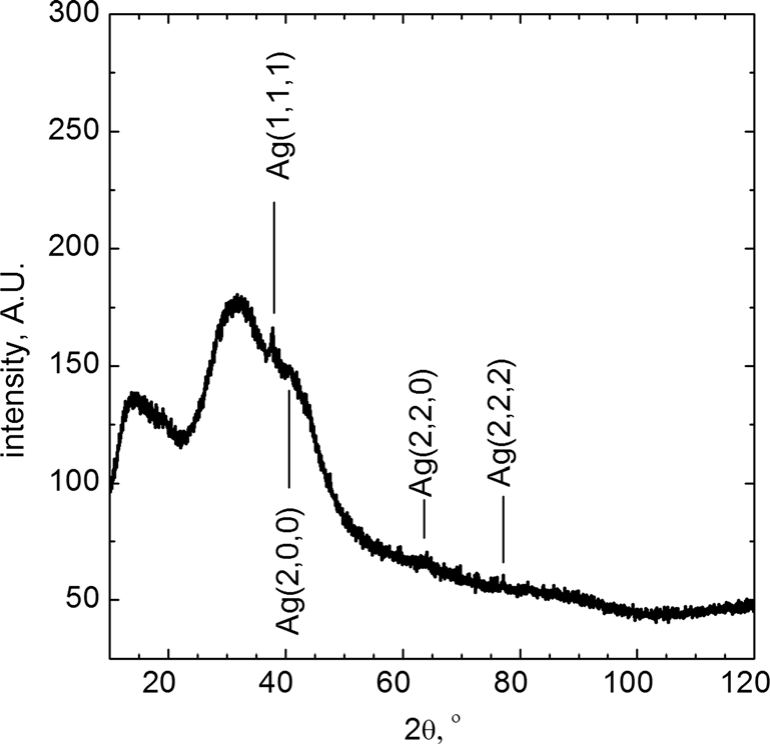
XRD pattern of obtained AgNPs

The results of further analyses of AgNPs carried out using XRD are shown in Fig. 5.

**Fig. 6:**
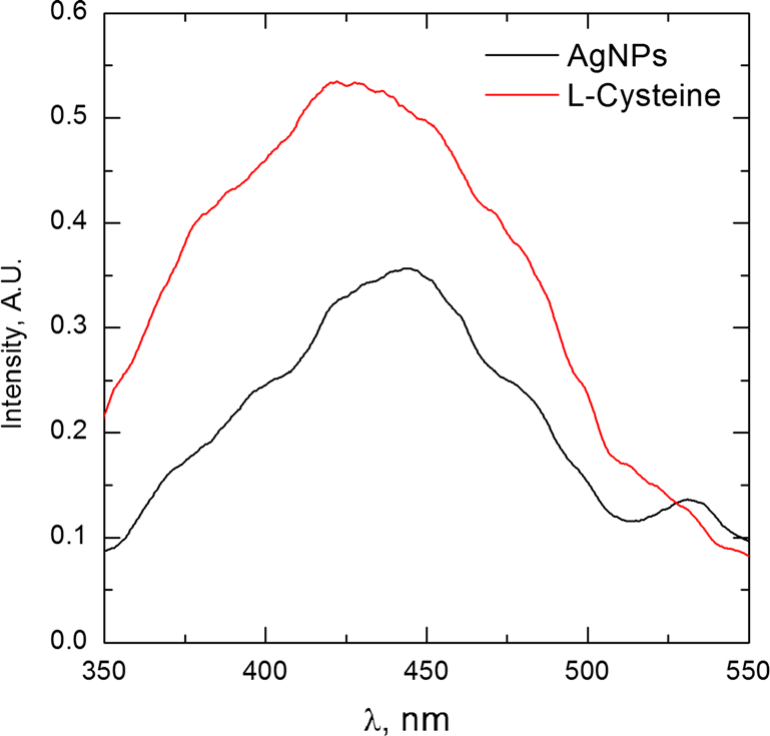
Fluorescence of AgNPs functionalized with l-cysteine

There is only one sharp peak. Low intensity of this peak is related to the particle size. As it was shown in Fig. 4a, obtained nanoparticles have polycrystal structure, where single grain size is significantly lower than 3 nm. Therefore, the peak broadening is observed which is also related to the Scherrer’s equation. Unfortunately, neither HR-TEM analysis nor XRD analysis confirmed the presence of Ag_2_S in the synthetized material. This might be related to the thickness of Ag_2_S layer. In the presence of silver sulfide on the surface of AgNPs, an additional optical effect should be observed, related directly to the semiconducting properties of Ag_2_S. That was the reason why the samples were also analyzed using fluorescence method. It is well known that silver sulfide exhibits quantum dots properties. Xiulan Wu et.al (2015) have shown that for spherical Ag_2_S nanoparticles of 5 nm in diameter, the maximum emission peak appears at the wavelength of ca. 750 nm, after excitation at 520 nm. It should also be underlined that Ag_2_S nanoparticles obtained by Xiulan Wu et al. were also functionalized using l-cysteine (Fig. 6).

As it can be seen from Fig. 6, the fluorescence of the sample is related to l-cysteine present on the surface of AgNPs. Insignificant changes in the left shoulder of the fluorescence curve of AgNPs are related to the absorption bend of metallic silver, as it is shown in Fig. 2a. There is no fluorescence above 600 nm. However, a small peak at 525 nm appears. This suggests that Ag-S bonding visible in XPS (see Fig. 1c) is related to the chemisorption of l-cysteine on the surface of AgNPs.

### Body weight changes in experimental animals

**Fig. 7:**
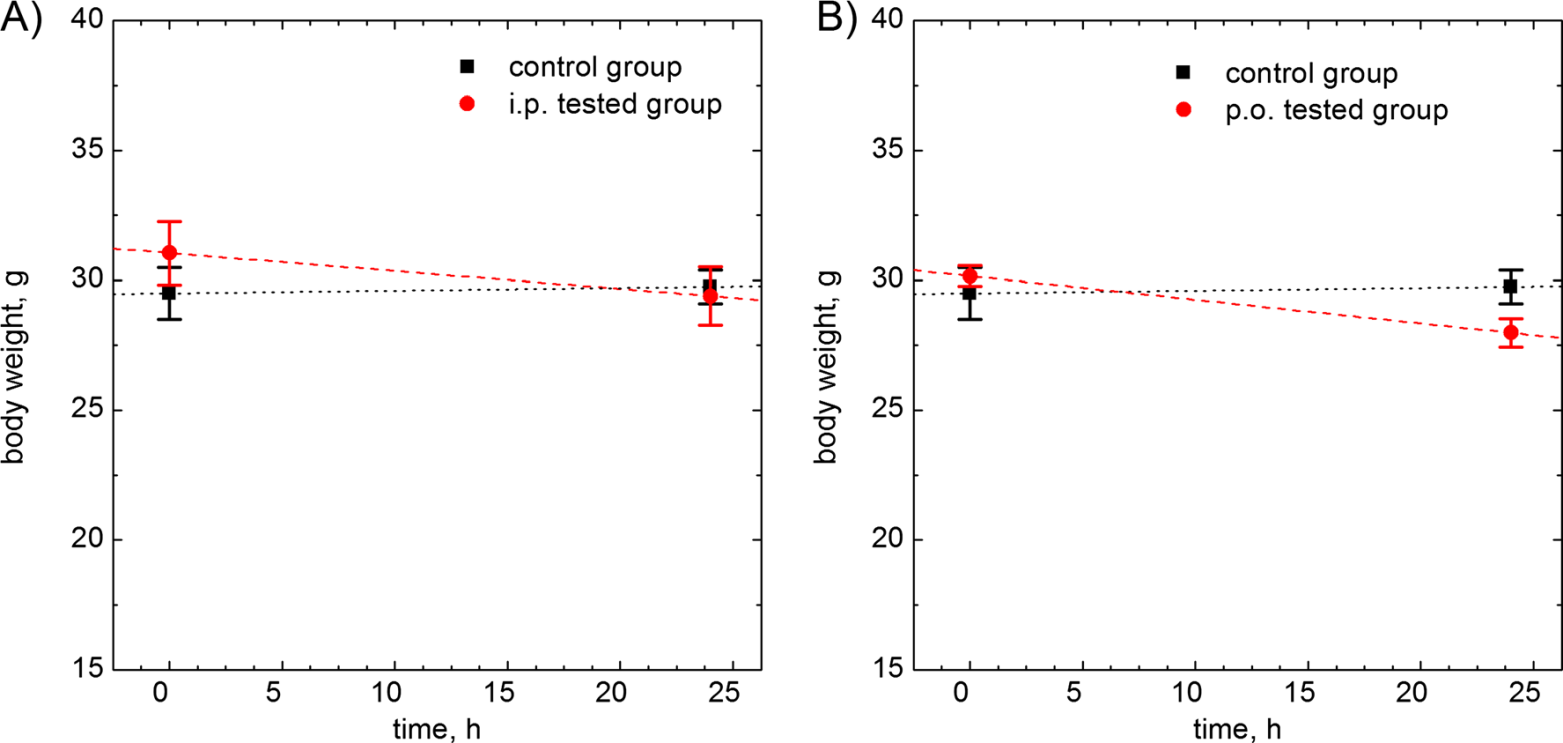
Body weight changes. **a** Comparison of control group to tested group after intraperitoneal administration. **b** Comparison of control group to tested group after intragastric administration. Mean ± SEM, *n* = 6. Statistical significance: *p < 0.05 (two-way ANOVA)

There were no significant changes in body weight of mice 24 h after intraperitoneal (i.p.) administration of the test solution in comparison to changes of body weight in mice from the control group (*p* = 0.0621). After intragastric (p.o.) administration, the changes of the body weight of tested mice were significantly lower in comparison to the body weight changes stated in mice (*p* = 0.0398). These results are shown in Fig. 7.

### AgNP acute toxicity and biodistribution

Acute toxicity is extremely important parameter for new drug safety. Thus, two ways of AgNP administration were chosen. The first one was intraperitoneal, and the second one was intragastric. During these trials, sedation of animals was noted. However, there was no death during the first 24 h. It can be concluded that synthesized AgNPs do not exhibit acute toxicity after intragastric and intraperitoneal administration.

Then, the mice were sacrificed and selected organs were removed for further analyses. The results of silver nanoparticles biodistributions are gathered and shown in Table 1.

**Table 1:** AgNP biodistribution in selected organs in percentage of given dose

Brain	Kidneys	Liver	Blood
After P.O administration			
1.82 × 10^−2^ ± 5.79 × 10^−3^	1.55 × 10^−2^ ± 2.21 × 10^−3^	6.53 × 10^−2^ ± 2.87 × 10^−2^	4.16 × 10^−2^ ± 1.51 × 10^−2^
After I.P administration			
3.59 × 10^−2^ ± 1.52 × 10^−3^	2.98 × 10^−1^ ± 6.24 × 10^−2^	2.93 ± 4.31 × 10^−1^	9.45 × 10^−2^ ± 5.46 × 10^−2^

The amount of AgNPs determined in selected organs is low. Only in the case of intraperitoneal administration, a significant accumulation in the liver was stated. The absorption from the digestive tract is very low. These results are comparable to our previous report (Bednarski et al. 2015). However, it should be stressed that the amount of AgNPs absorbed from the digestive tract is about two times higher in comparison to gold nanoparticles (Bednarski et al. 2015) .

### Irritating test

Additionally, an irritating test on the rate skin was performed. About 300 μL of AgNPs suspension was rubbed into the skin of rats (*n* = 3). After 24 h, no irritating effect was observed. Moreover, the presence of the silver in the blood was not detected. This in turn suggests that dermal absorption also does not occur, which indicates that AgNPs can be safely used in cosmetics.

## Conclusions

The antibacterial properties of silver nanoparticles are well known. In this paper, a simple and effective method for silver nanoparticle synthesis was described. These nanoparticles showed excellent biocompatibility. Thanks to the application of l-cysteine silver complex, it was possible to obtain the concentration of colloid equal to up to 10.7 g/L. Such a high concentration of colloid is extremely difficult to synthesize in one step. The only requirement for the developed method is the purification process, in order to remove leftover of reductant and l-cysteine.

Moreover, narrow particle size distribution of the colloid makes its behavior in living organism easy to investigate. Since the properties of nanoparticles depend on their size, in the case of polydisperse samples, the assessment of the biological effect can be fuzzy as different fractions can give different responses. Finally, it is worth mentioning that l-cysteine was applied as a stabilizing agent. This simple amino acid can be easily used to link an antibody or any other compound to the surface of nanoparticles.
